# Decreased Glucose Utilization Contributes to Memory Impairment in Patients with Glufosinate Ammonium Intoxication

**DOI:** 10.3390/jcm9041213

**Published:** 2020-04-23

**Authors:** Samel Park, Joong Il Kim, Nam-jun Cho, Se Won Oh, Jongkyu Park, Ik Dong Yoo, Hyo-Wook Gil, Sang Mi Lee

**Affiliations:** 1 Department of Internal Medicine, Soonchunhyang University Cheonan Hospital, Cheonan 31151, Korea; samelpark17@schmc.ac.kr (S.P.); chonj@schmc.ac.kr (N.-j.C.); 2 Future Medicine Division, Korea Institute of Oriental Medicine, Daejeon 34054, Korea; jikim@kiom.re.kr; 3 Department of Radiology, Eunpyung St. Mary’s Hospital, The Catholic University of Korea, Seoul 03312, Korea; oasis1979@gmail.com; 4 Department of Neurology, Soonchunhyang University Cheonan Hospital, Cheonan 31151, Korea; c89504@schmc.ac.kr; 5 Department of Nuclear Medicine, Soonchunhyang University Cheonan Hospital, Cheonan 31151, Korea; 92132@schmc.ac.kr

**Keywords:** herbicides, poisoning, memory disorder, positron emission tomography, glufosinate ammonium, F-18 flurodeoxyglucose

## Abstract

The symptoms of glufosinate ammonium (GLA) intoxication include gastrointestinal and neurologic symptoms, respiratory failure, and cardiovascular instability. Among these, neurologic symptoms including loss of consciousness, memory impairment, and seizure are characteristic of GLA poisoning. However, the mechanism of brain injury by GLA poisoning is still poorly understood. We investigated nine patients who had performed an F-18 fluorodeoxyglucose (FDG) positron emission tomography (PET) scan because of memory impairment caused by GLA ingestion. FDG-PET images of patients with GLA intoxication were compared with 24 age- and sex-matched healthy controls to evaluate whether the patients had abnormal patterns of glucose metabolism in the brain. Decreased glucose metabolism was observed in the inferior frontal and temporal lobes of these patients with GLA intoxication when compared with 24 age- and sex-matched healthy controls. Three patients performed follow-up FDG-PET scans. However, it was shown that the results of the follow-up FDG-PET scans were determined to be inconclusive. Our study showed that memory impairment induced by GLA intoxication was associated with glucose hypometabolism in the inferior frontal and temporal lobes in the brain.

## 1. Introduction

Glufosinate ammonium (GLA) is a structural analog of glutamate and acts by non-selectively inhibiting glutamate synthetase of plants, leading to the accumulation of ammonium ions and inhibition of photosynthesis [1]. Since the ban on the use of paraquat (bipyridylium) in Korea, intoxication by ingestion of GLA is increasing [2]. Because of differences in metabolic pathways between mammals and plants, GLA has been considered to be less toxic than other pesticides, such as paraquat, organophosphate, or carbamate [3]. However, several studies have reported the toxic symptoms induced by GLA ingestion, especially symptoms that involved the central nervous system, including neurologic symptoms such as loss of consciousness, seizure, and memory disturbance [4,5,6,7].

Despite the previous reports, the mechanisms by which GLA causes neurologic symptoms have not been well elucidated. However, given the results from previous studies, it is relevant that the N-methyl-D-aspartate (NMDA) receptor is involved in the mechanisms [8]. Because of the structural similarity between glufosinate and glutamate, glufosinate may bind to the NMDA receptors, which are the target for the glutamate that is normally present in a brain with various physiologic activities [8]. A previous study reported that hyperactivity of the NMDA receptor is associated with a glufosinate-induced seizure [9]. In addition, NMDA receptors are crucial for controlling synaptic plasticity associated with memory function [10]. Therefore, the pathway associated with NMDA receptors might have a causal relationship with memory loss by GLA ingestion. In a previous study, structural changes in the hippocampus and parahippocampal gyrus after GLA ingestion were reported [11]. In the mouse model, chronic exposure to GLA induces memory impairments and modification of hippocampal texture as demonstrated by magnetic resonance imaging (MRI) [12].

Although there are several studies suggesting a relationship between glufosinate and NMDA receptors, few studies have revealed the mechanism of memory loss after ingestion of glufosinate. An F-18 fluorodeoxyglucose (FDG) positron emission tomography (PET) scan is an imaging modality that can visualize glucose metabolism in a functional way. An FDG-PET study that evaluated the rate of regional glucose utilization revealed that a lower rate of glucose use is associated with cognitive dysfunction and memory impairment [13], therefore it is helpful for detecting early changes of the brain in neurodegenerative disorders, such as Alzheimer’s disease [14].

We retrospectively reviewed FDG-PET images in order to evaluate mechanisms of memory impairments in patients with GLA intoxication. To the best of our knowledge, this is the first study to examine the clinical significance of an FDG-PET scan in patients with GLA intoxication accompanied by memory impairment compared to normal healthy individuals.

## 2. Methods

### 2.1. Study Design and Participants

This study was designed to compare images of brain FDG-PET scans between patients with GLA intoxication and normal healthy subjects. Two expert nuclear medicine physicians screened the FDG-PET images obtained from the general population without any symptoms, which were performed just for medical checkup programs, from March 2012 to February 2018, because templates consisting of normal controls were needed in order to compare brain FDG-PET scans between the two groups, e.g., patients and normal healthy controls [15]. Among them, patients with images of brain FDG-PET scans were selected. Then, they were determined to be normal healthy subjects, when two expert nuclear medicine physicians and the program commercially provided by the vendor (Q brain, GE healthcare) interpreted that the brain FDG-PET scans showed no metabolic abnormalities. Eventually, a total of 164 patients were determined to be normal healthy subjects. After that, the images of nine patients were compared to the images of 164 patients. Among them, age- and sex-matched healthy controls were selected at a 1:3 ratio after pseudo-randomization, then compared to the patients with GLA intoxication to assess the alteration in glucose metabolism. When we were going to analyze the follow-up image, images of all patients with the same age range and gender among 164 patients were compared to the patient.

We screened patients who had been admitted to the Nephrology Department of Soonchunhyang University Cheonan Hospital (Cheonan, Korea), because of intentional oral intoxication of pesticide and/or general medicine, from October 2016 to September 2017. Among them, the patients who were intoxicated with GLA and had performed FDG-PET scans were retrospectively reviewed. We extracted data on all the patients who had performed MRI and FDG-PET scans because of symptoms of memory impairment, including both retrograde and anterograde amnesia. In the emergency room, because the patients showed unconsciousness, whether the patients had ingested GLA was identified based on the witness’s statement, pesticide bottles found with the patient, and the examination of remaining amounts of pesticide in the bottles by residents living with the patient or by neighbors. The exact amount of ingested volume was asked after the patients recovered.

The patients with memory impairment performed MRIs to exclude those with underlying anatomical lesions that could be associated with memory impairment including old infarctions and cerebromalacia. The patients with known cognitive dysfunction or dementia, of age above 85 years, or who had co-ingested psychiatric drugs that might influence the PET image were excluded in the analysis. The history of mild cognitive dysfunction or dementia, hypertension, diabetes mellitus, or any depression was investigated through interviews with patients or patients’ family members.

After discharge, patients were followed up via our outpatient department (OPD). During the visit, we interviewed patients thoroughly to evaluate the degree of memory dysfunction and whether there had been any recovery. They were followed up at OPD without any medications, because there is no evidence to recommend prescriptions for recovery from memory dysfunction induced by GLA.

This study was reviewed and approved by the Institutional Review Board of Soonchunhyang University Cheonan Hospital (Cheonan, Korea). It was conducted in accordance with the principles of the Declaration of Helsinki. The informed consents were waived because of its retrospective design (IRB-No: 2019-10-020).

### 2.2. MRI and FDG-PET Acquisition

All subjects underwent brain MRI scan to rule out underlying anatomical lesions, using a 3T MR scanner (Ingenia; Philips Healthcare, Best, the Netherlands). The imaging sequences included T1WI (repetition time/echo time (TR/TE), 500/10 ms), T2WI (TR/TE, 2314/80 ms), fluid-attenuated inversion recovery (FLAIR; TR/TE/TI, 9000/120/2500 ms), and T2*-weighted conventional gradient recalled echo images (TR, 710 ms; TE, 16 ms; flip angle = 18°). All images were obtained by 5 mm thickness and 22 × 22 cm field of view.

FDG-PET/CT scans were performed with a Biograph mCT 128 scanner (Siemens Healthineers GmbH, Henkestr, Erlangen, Germany). Patients were instructed to fast for at least 6 hours prior to the FDG-PET/CT scan, and all patients enrolled in the study showed a blood glucose level of less than 200 mg/dL before FDG administration. Approximately 370 MBq of FDG was administered intravenously 60 minutes before the start of FDG-PET/CT imaging. At first, a low-dose brain CT scan without contrast enhancement was done with 100 mA and 120 kVp. Afterward, FDG-PET imaging of the brain was done with an acquisition time of 10 minutes. FDG-PET images were reconstructed with a point-spread-function based Gauss and all-pass filter algorithm and time-of-flight reconstruction with attenuation correction.

### 2.3. FDG-PET Image Preprocessing and Voxel-Based Single-Subject Analysis

PET image analyses between the patients and healthy controls consisted of two processes. Firstly, we compared FDG-PET images of nine intoxicated patients with 164 healthy controls to evaluate whether the patients had an abnormal pattern of glucose metabolism in the brain. Then, from the 164 healthy subjects, age- and sex-matched healthy controls were selected at a 1:3 ratio after pseudo-randomization, because age and sex could impact on results of FDG-PET scans.

All image processing of FDG-PET scans obtained from healthy controls and patients were done using statistical parametric mapping (SPM8, Wellcome Trust Centre for Neuroimaging, UCL Queen Square Institute of Neruology, University College London, London, UK) implemented in MATLAB (version 2016b, MathWorks, Inc., Natick, MA, USA). Spatial preprocessing of FDG-PET images used the following procedure. First, all image data obtained in Digital Imaging and Communications in Medicine (DICOM) format was converted to Neuroimaging Informatics Technology Initiative (NIFTI) format for further image processing and analysis [16]. Second, each image was spatially normalized to Montreal Neurological Institute (MNI) space defined by the PET template provided from SPM with the following parameters: no source/template weighting, source image smoothing 8 mm, no template image smoothing, affine regularization to International Consortium of Brain Mapping (ICBM)/MNI, nonlinear frequency cutoff 25, nonlinear iterations 16, nonlinear regularization 1, preserve concentrations, bounding box [−90 −126 −72; 90 90 108] with 2 mm isotropic voxel size and trilinear interpolation [17]. Third, each normalized FDG-PET image was subsequently smoothed by convolution with an isotropic 3D Gaussian kernel of 6 mm full width at half maximum (FWHM) [18,19].

After image preprocessing, spatial normalized FDG-PET image intensities were scaled in proportion to the overall mean (global mean scaling) to minimize inter-subject variability. To evaluate hypometabolism of a patient compared to healthy controls, voxel-based single-subject analysis was carried out using the two-sample t-test. Second, for the patients who had undergone follow-up FDG PET/CT images, individual patient analysis was performed to assess the changes of brain glucose metabolism during the follow-up period.

## 3. Results

**Figure 1:**
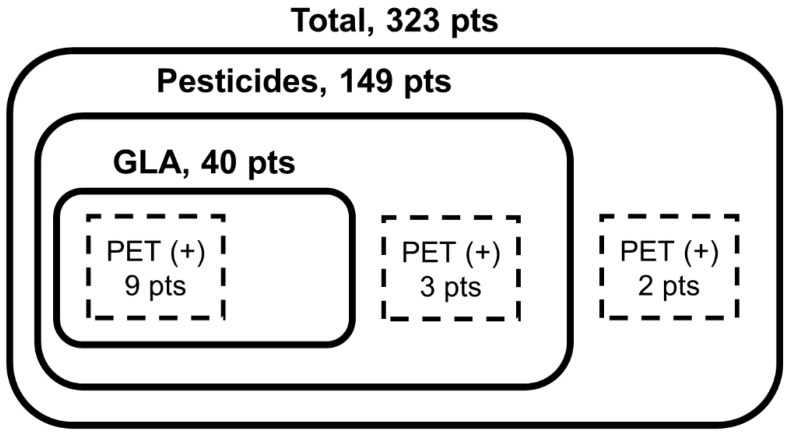
A schema to illustrate the selection process of patients. GLA, glufosinate ammonium; PET, positron emission tomography.

A total of 323 patients were admitted to our center because of intoxication from pesticides and/or general medications during the period. Among them, 40 patients reported GLA intoxication, and 149 patients were confirmed as having ingested pesticides other than GLA. Among a total of 323 patients, 14 patients performed FDG-PET scans. Among them, three patients were excluded because they had ingested pesticides other than GLA (two patients, glyphosate; the other, chlorophenoxy). One other patient was excluded because of having ingested psychiatric drugs, and another patient because of both a history of dementia and ingested psychiatric drugs. Eventually, nine patients who ingested GLA were involved (Figure 1). All nine patients and their family members denied a previous history of dementia or memory impairment. These patients performed FDG-PET scans 13 (range: 4–104) days after GLA intoxication, because they continuously complained of memory impairment after enough time had passed for acute toxic symptoms to disappear. All patients consulted with a neurology department because of memory impairment and/or a seizure and had no significant anatomical findings documented by the brain MRI.

The baseline clinical characteristics are presented in Table 1. Among the patients, seven patients experienced a seizure. Six patients had results from the mini-mental state examination (MMSE) and clinical dementia rating (CDR) test. All patients complained of memory impairment after recovery of consciousness, including both anterograde and retrograde amnesia. For example, they could not remember the breakfast menu of the day. The caregivers often complained that a patient could not even remember what had happened just before, suggesting symptoms associated with anterograde amnesia. Family members noticed that the patient’s autobiographical memories, including the patient’s own job, family members or friends, and personal experiences, such as travel to special place, the birth of one’s grandchildren, or marriage of their descendants, were lost. That is, the patients experienced both retrograde and anterograde amnesia simultaneously.

**Figure 2:**
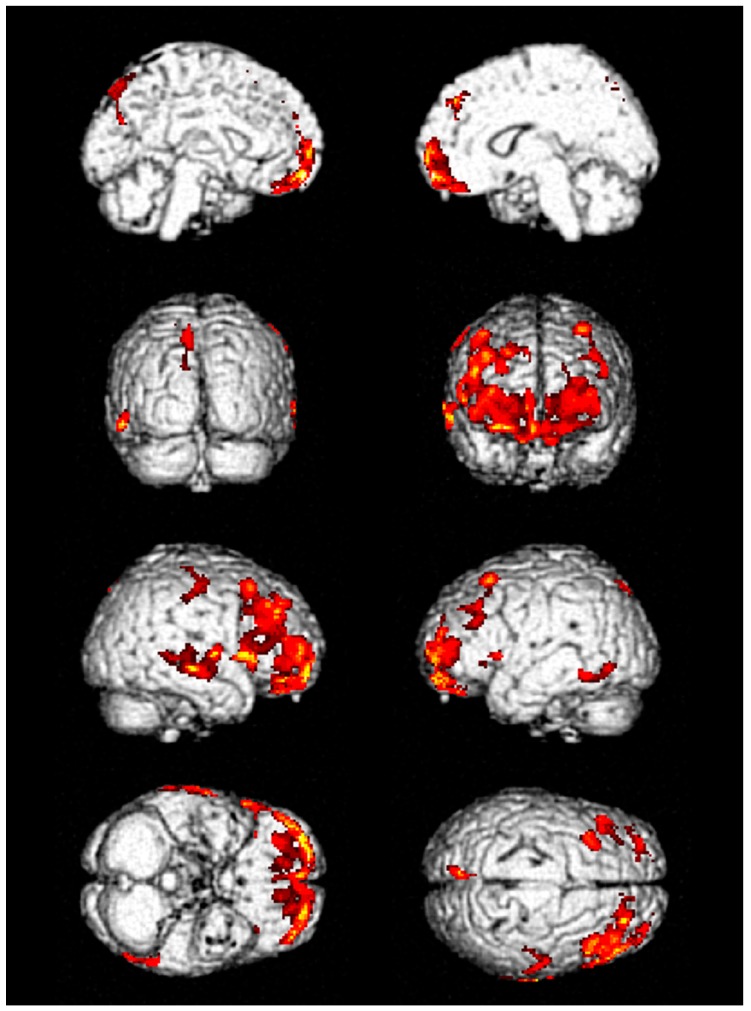
The results of group analysis of brain FDG PET/CT. Three age- and sex-matched patients were selected from the healthy subject group at a 1:3 ratio after pseudo-randomization and then compared to the patients with glufosinate ammonium intoxication to assess the alteration in glucose metabolism. The areas colored with red in the figure showed decreased FDG uptake in PET images of nine patients compared to the PET images of matched healthy control subjects. Nine intoxicated patients revealed significantly decreased glucose metabolism in the frontal and temporal cortex.

In the comparison between the patients and normal healthy controls, decreased glucose metabolism was observed in the frontal, especially inferior frontal, and temporal lobes of the patients with GLA intoxication (Supplemental Figure S1 and Supplemental Table S1). These alterations were retained when compared to age- and sex-matched healthy control group after pseudo-randomization (Table 2, Figure 2, and Supplemental Table S2). Among them, eight patients were able to follow up for 1–12 months via OPD, and three patients performed follow-up FDG-PET scans.

**Figure 3:**
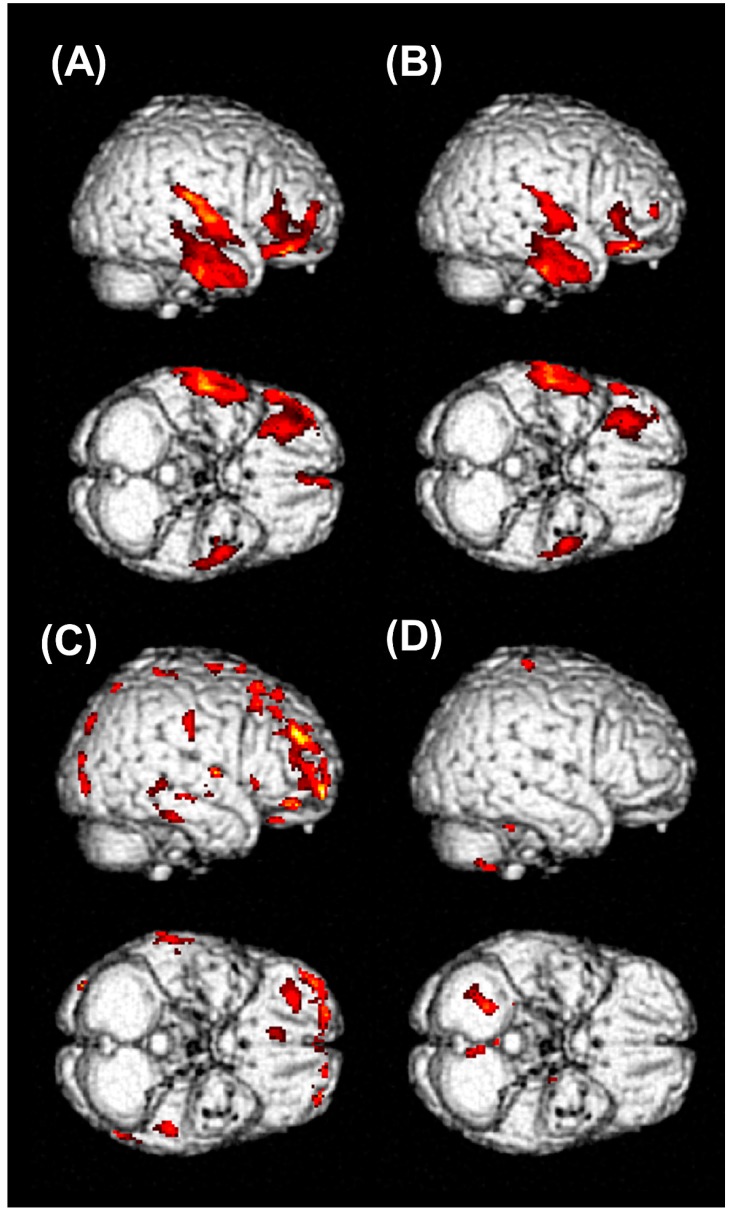
The result of individual analysis of brain FDG-PET/CT. The area colored red in the figure showed decreased FDG uptake in PET images of patients compared to the those of age- and gender-matched healthy control group. (**A**) The results of an initial brain FDG-PET in a 67-year-old man showed decreased glucose metabolism in an inferior area of the right frontal cortex and right temporal cortices. (**B**) On follow-up brain FDG-PET images 6 months after intoxication, decreased glucose metabolism in the right frontal and temporal cortices was persistently observed. (**C**) The results of an initial brain FDG-PET in a 57-year-old man showed decreased glucose metabolism in the right frontal cortex. (**D**) On 2 months follow-up brain FDG PET images, decreased glucose metabolism in the right frontal cortex was almost recovered. (**A**,**B**) Compared to the age of approximately 60 years and male normal healthy subjects (*n* = 16). (**C**,**D**) Compared to the age of approximately 50 years and male normal healthy subjects (*n* = 16).

The patient who had longest period of admission and ICU stay days was followed-up for one year after intoxication. He lost his memory for his job, colleagues, and some personal experiences after GLA intoxication. The symptoms associated with anterograde amnesia, which showed up at the early period of hospitalization, improved several months later after discharge from the hospital. However, most of the lost memories were not recovered, even after a year. He had a history of hypertension and diabetes, but not a history of dementia or cognitive dysfunction. His family members described that his cognitive and memory function had been completely normal and that he had normal socioeconomic activity as an engineer before the intoxication by GLA. The follow-up FDG-PET scans were performed at 4, 6, and 12 months after intoxication. A severely decreased glucose metabolism that was noted in the inferior area of frontal lobes and temporal lobes remained after significant time had elapsed from the GLA intoxication, raising concerns that it could cause irreversible damage to the brain (Figure 3A,B).

In the last two patients who had a follow-up scan, the symptoms associated with anterograde amnesia disappeared during the OPD follow-up. The examples of baseline and follow-up FDG-PET images in one of those two patients are shown in Figure 3C,D. Decreased glucose metabolism in the frontal cortex had almost recovered at his 2 months follow-up FDG-PET scan (Figure 3D); nevertheless, retrograde amnesia, especially episodic autobiographical memory loss, remained.

In the remaining six patients, who did not have follow-up FDG-PET scans, the anterograde amnesia improved. However, only partial restoration of lost old autobiographical memory could be observed.

## 4. Discussion

In the present study, we found retro- and anterograde amnesia accompanied by decreased glucose metabolism, especially in the inferior frontal and temporal lobes, in the patients with GLA intoxication, although significant anatomical lesions were not documented on a brain MRI. Our results suggested an association between memory impairment and decreased glucose metabolism within the patient’s brain with GLA intoxication. Notably, time-lags between intoxication events and the PET-scan acquisition imply that GLA had a chronic, long-lasting effect on memory impairments, not just an acute effect. To the best of our knowledge, this is the first study that has demonstrated the declines in glucose metabolism of the brain in patients with GLA intoxication using FDG-PET scans.

Many studies show that occupational or environmental exposure to pesticides is associated with neuropsychological impairments, including cognitive function, Parkinson’s disease, and Alzheimer’s disease [20,21,22,23,24]. However, there has been no long-term follow-up study in patients with oral pesticide intoxication. Small case series and studies have reported symptoms associated with GLA intoxication, such as loss of consciousness, seizure, irritability, memory impairment, respiratory deterioration, hypotension, hyperammonemia, rhabdomyolysis, and metabolic acidosis [5,6,7,11,25,26].

Watanabe et al. have reported the first report on neurologic effects of GLA intoxication, in which the patients complained of retro- and anterograde amnesia as our patients did [4]. Park et al. have reported a case with GLA intoxication. In that case, the patient showed a symmetrical high signal intensity in the hippocampal and parahippocampal area [11], implying that the bilateral hippocampal area might be responsible for anterograde amnesia in patients intoxicated with GLA. In our study, all patients lost autobiographical memory. Some of those lost their memory for several years. The results of MMSE from six patients implied decreased ability to recall past memory. When the patients were asked about the breakfast menu of that day, none of them could answer. During a brief follow-up through the OPD, we found that lost memory in most patients was incompletely recovered. We could not precisely evaluate the degree of recovery of the patient’s lost memory and how much the memory function improved with a proper tool. However, the family members stated that they could not recall a few past memories that the patients should naturally remember. The details of memory impairment in our patients also suggested a relationship between GLA intoxication and hippocampal lesion. In addition, in our most severe case, unrecovered lesions remained in the PET CT after sufficient time (Figure 3). This might suggest the possibility of irreversible change induced by GLA.

An FDG-PET scan is an effective image modality to improve the diagnosis of dementia and predict progressive cognitive impairment [27,28]. Figure 2 shows decreased glucose metabolism in the inferior frontal and temporal lobes of the patients with GLA intoxication compared to age- and sex-matched normal healthy controls. In Alzheimer’s disease, the most common cause of dementia in the elderly [29], FDG-PET demonstrates typical patterns of hypometabolism in the parietotemporal cortices, posterior cingulate gyrus, and precuneus areas [30]. However, the FDG-PET images in patients with GLA intoxication showed significant changes in frontotemporal cortices, comparable to the patterns of frontotemporal dementia (FTD), which shows hypometabolism in the frontal and anterior temporal lobe [30]. A previous PET study has shown that retrieval mechanisms for autobiographical memory were deteriorated in frontal variant FTD. Indeed, inferior frontal and medial temporal regions contribute to the retrieval of autobiographical memory [31].

In addition, it was intriguing that seizure and memory impairment were identified simultaneously. Since GLA causes seizures through the NMDA receptor [9], it could be assumed that memory impairment occurs that way also. It is considered that blocking the NMDA receptor induces memory impairments and the associated mechanism could be a target of therapy for Alzheimer’s disease [10]. Ketamine, an NMDA receptor antagonist, induces cognitive and memory impairments dose-dependently [32]. However, activation of the NMDA receptor has a pathophysiologic role in seizures, and the NMDA receptor antagonist has been considered to have an antiepileptic effect [33].

Although these paradoxical and opposing mechanisms should be evaluated in future studies, the diminishment of increased glucose metabolism by an NMDA receptor antagonist in rats could be a putative mechanism to explain our results [34]. Glutamate is an excitatory neurotransmitter, which affects neuronal activity via several ionotropic and metabotropic receptors in the brain [35]. Glutamate released from excitatory synapses increases glucose metabolism in astrocytes coupled with neuronal activity, which is visualized as glucose hypermetabolism in brain FDG-PET scans [36]. A recent study showed that activation of glutamate transport in astrocytes triggered glucose uptake in the rodent brain and that astrocytes contribute to the FDG-PET signals [37]. Therefore, if GLA blocked the glutamate receptor on the astrocyte, not the neuron, it could theoretically affect net-glucose metabolism, resulting in the decreased glucose metabolism demonstrated on FDG-PET scans. In comparison, neuronal hyperactivity of the glutamate receptor caused by GLA might explain the pathogenesis of the seizure that appeared concurrently with memory dysfunction. The memory encoding process, called long-term potentiation, is associated with glutamate receptors, including NMDA and α-amino-3-hydroxy-5-methyl-4-isoxazolepropionic acid (AMPA) receptors, and plays a critical role in the formation of episodic memory [38], where the astrocyte–neuron lactate shuttle plays a key role [39].

In a previous study, epileptogenic foci were identified as hypometabolism in FDG-PET and hyperperfusion in ictal single-photon emission computed tomography [40]. Given previous studies that support evidence of glucose hypometabolism in epileptogenic foci in interictal FDG-PET [40,41,42], we supposed that the glucose hypometabolism demonstrated in our patients might be associated with a seizure, which seven of the nine patients had experienced. In this respect, a patient with the most severe symptoms (who also had the most extended hospital and ICU stay days) suffered from a sustained seizure until hospital day 6, whereby there was performed FDG-PET CT 98 days after the seizure stopped. In other patients, the seizure was noted to have stopped within 72 hours after ingestion, similar to our previous report [7], and FDG-PET images were obtained 7–58 days after the seizure had not occurred. Although further research on seizure and glucose hypometabolism, caused by GLA, could help reveal the mechanism of its neurotoxicity, in this study, the effect of seizure on glucose metabolism might not be a significant factor.

Our study had several limitations. First, this was a retrospective observational study. Notably, before the index patient who had severe memory impairment after GLA intoxication, we did not focus on the GLA-induced anterograde amnesia. The experience of the index patient who did not recover several days after led us to evaluate PET scans in patients with GLA intoxication. This is why one patient had PET scans 104 days after the intoxication event and why the intervals between the ingestion of GLA and the acquisition of FDG-PET were heterogeneous. Additionally, he experienced the most severe toxic symptoms and had the longest hospital and ICU stay days among our patients in this study. These conditions might contribute to a long-lasting decline in glucose metabolism noted in the brain FDG-PET scan. Furthermore, the patients were followed without prespecified protocols because we did not have any follow-up data on such GLA-induced memory impairment. Despite these limitations caused by retrospective design, our results could be a cornerstone for a prospective observational study. In addition, this study was not designed to follow-up patients prospectively. Instead, comparing the result of FDG-PET scans between the patients with memory impairment induced by GLA and healthy control cross-sectionally was the main focus of this study.

Second, there might be a selection bias in determining who performs FDG-PET scans, affecting the results. However, among 40 patients who ingested GLA, nine patients performed FDG-PEG scan, comparing, just three patients performed FDG-PET scans among 189 patients intoxicated with other pesticides during the same period. Although considering that this was a retrospective study, the result may not be caused by coincidence.

Third, the small number of patients could be problematic if applied to the general population. To overcome this shortcoming, we compared our patients with age- and sex-matched normal healthy population. The healthy population performed brain FDG-PET scans for general health check-up and were approved as “normal for age” by two specialists of nuclear medicine. This process could improve the reliability of our research.

Fourth, the methods of evaluating the degree of cognitive dysfunction and memory loss were neither objective nor quantitative. In order to confirm whether the hypometabolism observed in the FDG-PET scans correlated with the actual cognitive decline, detailed cognitive assessment of each area was needed. However, patients did not want to do more tests, including such time-consuming tests. Instead, we tried to assess the patient’s current and updated status every time we were meeting patients. We asked the patients to remember what they ate that day to evaluate whether they could recall the menu of breakfast or lunch one by one. We also proposed to the patients to have them memorize three or four words and to recall the words after a few minutes later, which is a test similar to the MMSE. We checked whether they could remember the most important event in their lives, such as the wedding event of the patient’s descendants. In addition to these subjective characteristics, we confirmed that the patients had no anatomic lesions contributing to the condition of cognitive dysfunction, with documentation of any noted abnormality of glucose metabolism.

## 5. Conclusions

Our study showed that memory dysfunction induced by GLA intoxication, including anterograde and retrograde amnesia, was associated with glucose hypometabolism in the brain, especially in the inferior frontal and temporal lobes, without significant anatomical lesions. In addition, our results also shed light on the pathogenesis of GLA-induced memory loss and on the specific lesion associated with a mechanistic role.

## Figures and Tables

**Table 1:** Clinical characteristics of patients who suffered from memory impairment associated with glufosinate ammonium intoxication (*n* = 9).

Variables	Values
Male sex, n (%)	5 (55.6%)
Age, year	57 (38–83)
Hypertension, n (%)	3 (33.3%)
Diabetes mellitus, n (%)	1 (11.1%)
Depression, n (%)	2 (22.2%)
Current smoker, n (%)	4 (44.4%)
Alcohol history, bottles/week	1 (0–14)
^†^ Ingested volume, mL	300 (100–500)
Seizure, n (%)	7 (77.8%)
^†^ Initial GCS, score	9 (3–15)
^†^ Initial ammonia, mg/dL	130.6 (69.1–230.0)
^†^ MMSE (*n* = 6)	24.5 (14–28)
^†^* Orientation (10)	8.5 (6–10)
^†^* Registration (3)	3 (0–3)
^†^* Attention and Calculation (5)	3 (0–4)
^†^* Recall (3)	2.5 (0–3)
^†^* Language (9)	9 (6–9)
^†^ CDR (*n* = 6)	0.5 (0–0.5)
APACHE II score	20 (6–35)
Systemic hypotension, n (%)	6 (66.7%)
Acute kidney injury, n (%)	1 (11.1%)
Mechanical ventilation, n (%)	7 (77.8%)
^†^ Hospital day	12 (6–53)
^†^ ICU stay day	6 (3–41)

^†^ Continuous variables were presented as median (range); * Components of MMSE. Abbreviations: GCS, Glasgow coma scale; MMSE, mini-mental state examination; CDR, clinical dementia rating; APACHE II, acute physiology and chronic health evaluation II; ICU, intensive care unit.

**Table 2:** The results of the F-18 fluorodeoxyglucose positron emission tomography (FDG-PET) for each patients and clinical information, including hospital days, ICU stay days, seizure duration, and follow-up duration.

No.	FDG-PET Finding			Hospital Stay, Days	ICU Stay, Days	Seizure Duration, Days	F/U Duration, Months
	Initial	Follow up (I)	Follow up (II)				
1	Decreased metabolic activity in frontal and temporal lobes	Improved but remained	Improved but remained	53	41	6	12
2	Decreased metabolic activity in frontal and temporal lobes	Improved	Improved	6	3	No	3
3	Decreased metabolic activity in frontal and temporal lobes	Not performed	Not performed	14	7	2	6
4	Decreased metabolic activity in inferior frontal lobe	Not performed	Not performed	10	6	1	1
5	Decreased metabolic activity in frontal lobe	Not performed	Not performed	12	4	1	1
6	Decreased metabolic activity in frontal and temporal lobes	Not performed	Not performed	24	8	3	2
7	Decreased metabolic activity in frontal and temporal lobes	Not performed	Not performed	19	13	3	1
8	Decreased metabolic activity in frontal and temporal lobes	Improved	Not performed	9	3	No	2
9	Decreased metabolic activity in frontal and temporal lobes	Not performed	Not performed	11	5	2	Loss

## Supplementary Materials

The following are available online at https://www.mdpi.com/2077-0383/9/4/1213/s1, Figure S1: The results of the group analysis of brain FDG PET/CT with a comparison between patients with glufosinate ammonium intoxication and 164 normal healthy subjects, Table S1: The results of statistical parametric mapping (SPM) analysis of brain FDG PET/CT between nine patients with glufosinate ammonium intoxication and 164 normal healthy subjects, Table S2: The results of SPM analysis of brain FDG PET/CT between nine patients with glufosinate ammonium intoxication and age- and sex-matched normal healthy subjects at a 1:3 ratio after pseudo-randomization.
