# Supplementary material for: Decreased Glucose Utilization Contributes to Memory Impairment in Patients with Glufosinate Ammonium Intoxication
Source: J Clin Med. 2020 Apr 23;9(4):1213. doi: 10.3390/jcm9041213 (PMC7231126; doi:10.3390/jcm9041213)
Supplement: Supplementary file 1 [file jcm-09-01213-s001.pdf]

Supplementary materials: Decreased Glucose Utilization Contributes to Memory Impairment in Patients with Glufosinate Ammonium Intoxication

Samel Park [1,†], Joong Il Kim [2,†], Nam-jun Cho [1], Se Won Oh [3], Jongkyu Park [4], Ik Dong Yoo [5], Hyo-Wook Gil [1,*] and Sang Mi Lee [5,*]

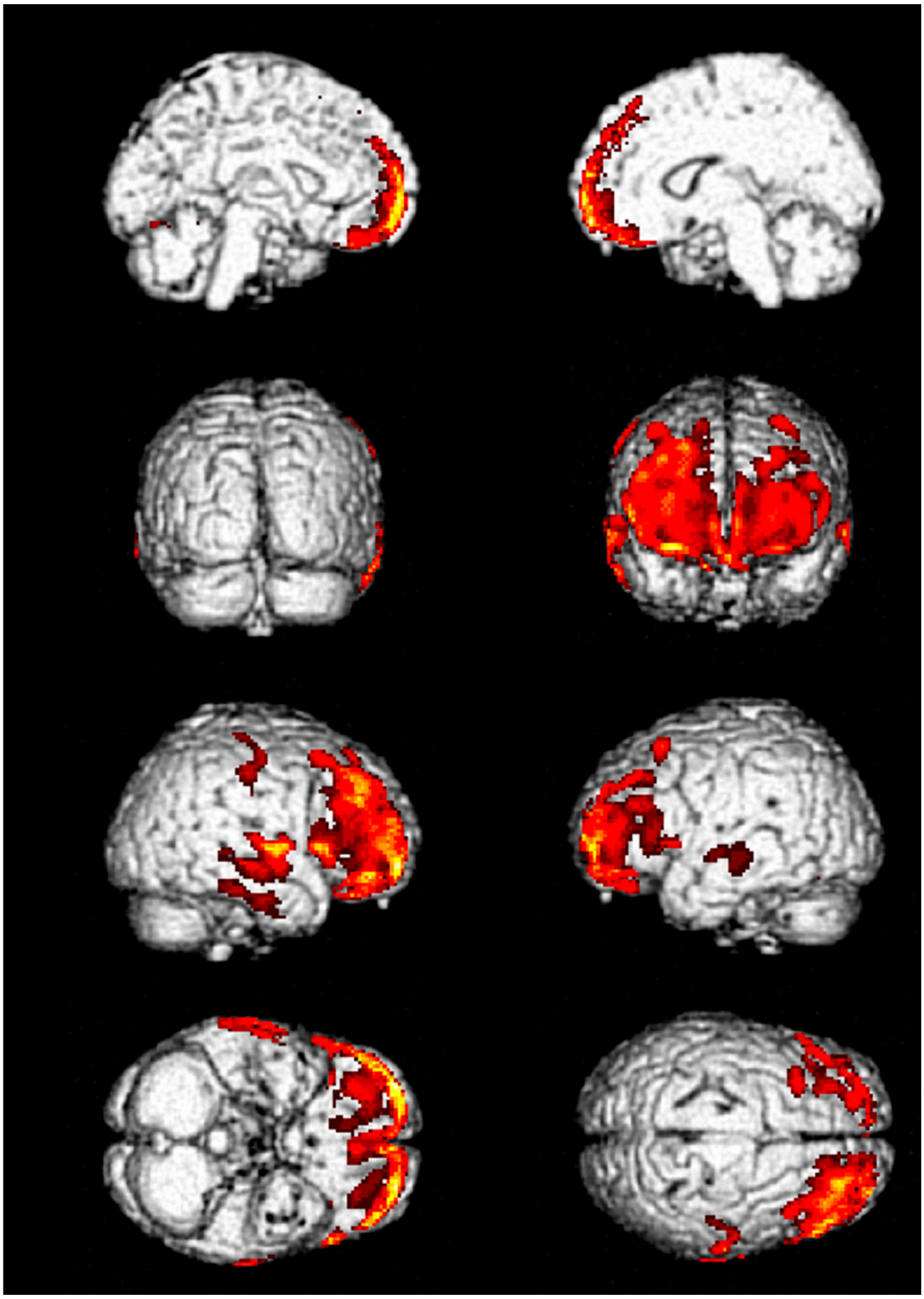

Figure S1. The results of the group analysis of brain FDG PET/CT with a comparison between patients with glufosinate ammonium intoxication and 164 normal healthy subjects. The areas colored red in the figure showed decreased FDG uptake in PET images. Significantly reduced glucose metabolism in the frontal and temporal cortex was observed in nine intoxicated patients.

Table S1. The results of statistical parametric mapping (SPM) analysis of brain FDG PET/CT between nine patients with glufosinate ammonium intoxication and 164 normal healthy subjects.

Location	BA	kE	T-value	MNI coordinates (mm)		
				X	Y	Z
Left/Right Superior/Middle Frontal Gyrus	9/10	7199	8.742	30	60	−6
			8.143	−22	60	0
			8.116	40	36	34
Left Insula	13	113	6.830	−34	18	4
Left Superior Frontal Gyrus	8	114	6.565	−28	20	56
Right Fusiform Gyrus	20/37	150	6.433	62	−12	−28
Right Inferior Temporal Gyrus			6.252	68	−32	−20
			5.678	58	−6	−34
Right Postcentral Gyrus	3/4	146	6.061	62	−20	46
			5.902	50	−26	64
			5.818	56	−14	52
Left Fusiform Gyrus	20/37	109	5.762	−24	−66	−14
Left Inferior Temporal Gyrus			5.745	−24	−52	−14
Left Lingual Gyrus	18/19		5.376	−18	−74	−12
Left Middle Temporal Gyrus	21	110	5.686	−66	−22	−12
			5.679	−62	−10	−4

The thresholded *p*-value was < 0.05 (FWE corrected) and cluster size, kE was > 100 voxels. Abbreviations: MNI coordinate, stereotaxic coordinate system of human brain from Montreal Neurological Institute; BA, Brodmann Area; FEW, familywise error rate; kE, cluster extent size.

Table S2. The results of SPM analysis of brain FDG PET/CT between nine patients with glufosinate ammonium intoxication and age- and sex-matched normal healthy subjects at a 1:3 ratio after pseudo-randomization.

Location	BA	kE	T-value	MNI coordinate (mm)		
				X	Y	Z
Right Superior Frontal Gyrus	9	4259	9.326	28	40	28
	8		8.079	40	20	54
			7.939	40	36	36
Left Superior Frontal Gyrus	8	137	8.517	−32	16	56
			5.065	−36	20	48
Left Inferior Temporal Gyrus	20/37	133	7.843	−52	−68	−6
			6.946	−56	−50	−12
Left Precuneus	7	150	6.757	−6	−74	52
			6.490	−10	−82	48
			6.081	−10	−72	26
Left Superior/Middle Frontal Gyrus	9/46	148	6.672	−46	30	26
			6.050	−40	28	38
			5.237	−30	40	36
Left Insula	13	102	6.410	−40	14	2
			5.404	−32	26	−2
Right Postcentral Gyrus	3	128	6.196	60	−18	50
			5.427	56	−12	56
			5.407	48	−26	66

The thresholded *p*-value was < 0.001 (FDR corrected) and cluster size, kE was > 100 voxels. Abbreviations: MNI coordinate, stereotaxic coordinate system of human brain from Montreal Neurological Institute; BA, Brodmann Area; FDR, false discovery rate; kE, cluster extent size.
